# Supplementary material for: Downscaling local distribution of cattle over Guadeloupe archipelago: An adapted method for disaggregating census data
Source: PLoS One. 2026 Jan 21;21(1):e0324695. doi: 10.1371/journal.pone.0324695 (PMC12822920; doi:10.1371/journal.pone.0324695)
Supplement: S1 File — Figures and tables of this document are referenced in the text as S1 to S7 in this manuscript. (PDF) [file pone.0324695.s001.pdf]

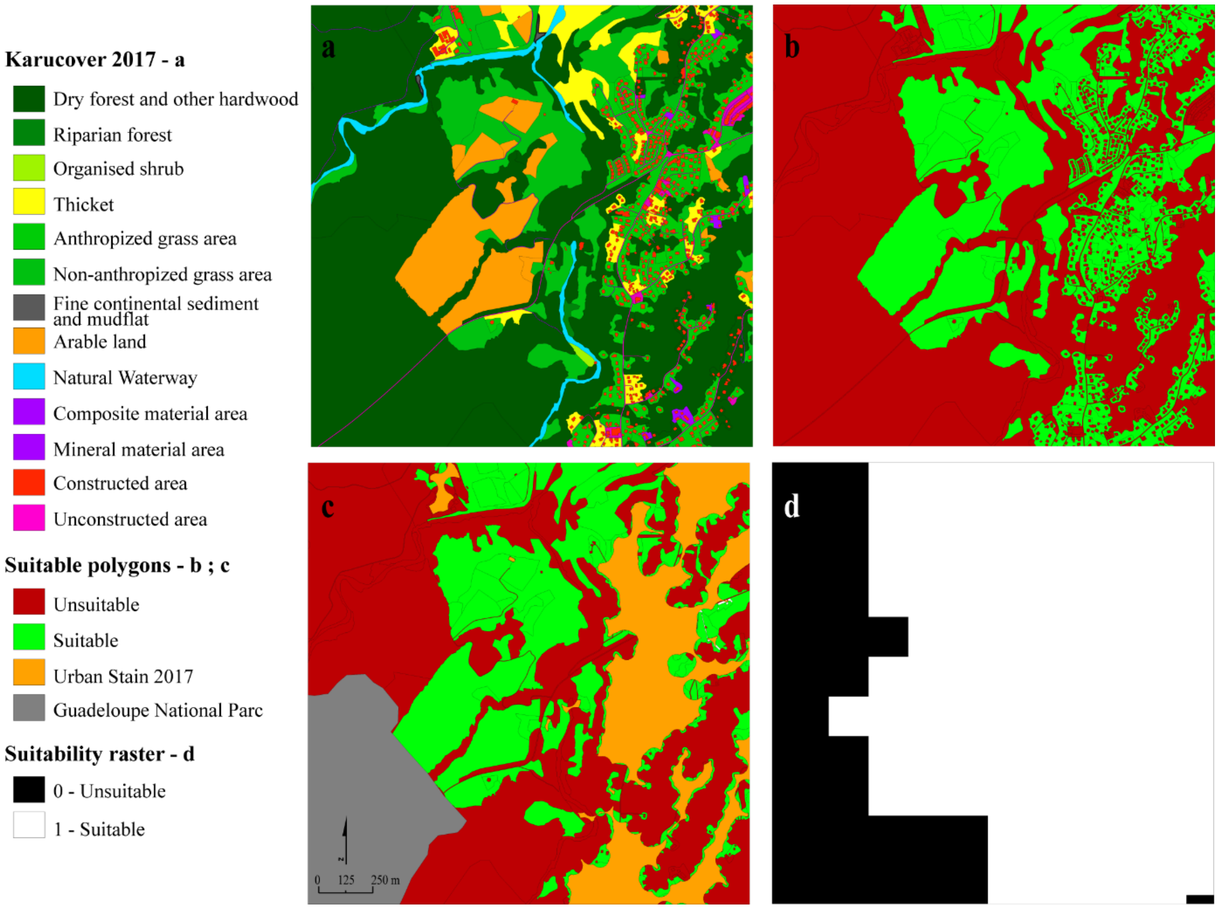

S1 Figure. Suitability raster creation processes (Zoom on the municipality of Petit-Bourg (Prise d'eau sector, Guadeloupe). Created by Victor Dufleit with data from Karucover 2017 dataset (Robillard 2022; [24] in manuscript), which are in the public domain.

S2 Table. Masks used to build predictions maps and to create the suitability raster.

Variable Name	Type	Use	Source
Guadeloupe Administrative boundary	Land	Land Mask	BD TOPO®
Karucover Suitability derived raster	Land use	Suitability Mask	Karugéo
National Parc polygon	Land use	Construction Mask	BD TOPO®
Guadeloupe "urban stain" 2017	Land use	Construction Mask	Karugéo

S3 Table. Symplification of level 5 Karucover 2017 land cover classes

KaruCover_2017 Cover Class level 5	New Cover Class
Zone bâtie	No vegetation
Zone non bâtie	No vegetation
Zone à matériaux minéraux – pierre-terre	No vegetation
Zone à autres matériaux composite (décharges…)	No vegetation
Sédiment fin continental et vasière	No vegetation
Plage de sable	No vegetation
Pierre	No vegetation
Rocher	No vegetation
Voie d’eau naturelle	Wetland
Canal	Wetland
Plan d’eau	Wetland
Bassin d’eau maritime	Wetland
Autre eau maritime	Wetland
Mangrove	Wetland
Forêt marécageuse	Wetland
Ripisylve	Forest
Forêt littorale	Forest
Forêt sèche et Autres feuillus	Forest
Marais	Wetland
Formation arbustive organisée	Arable land
Fourré	Thicket
Espace herbacé non anthropisé	Grass
Espace herbacé anthropisé	Grass
Terre arable	Arable land

S4 Table. Symplification of level 5 Karucover 2017 land use classes

KaruCover 2017 Use Class	New Use Class
Aérien	Unsuitable
Autre espace agricole	Agricultural
Autre infrastructures sportive	Other
Autre service de loisirs	Other
Autre service publique	Unsuitable
Autre usage résidentiel	Other
Bananeraie	Culture
Bâtiment agricole et espaces associés	Agricultural
Canne à sucre	Cropland
Carrière	Unsuitable
Centre équestre	Other
Commerce	Unsuitable
Déchet, traitement et recyclage	Unsuitable
Délaissé urbain et friche d'activité économique	Other
Eau et traitement de l'eau	Unsuitable
Éducation	Unsuitable
Élevage	Breeding
Espace agricole non exploité	Agricultural
Golf	Unsuitable
Grand collectif mixé	Dense housing
Grand collectif non mixé	Dense housing
Habitat continu dense mixé à d'autres usages	Dense housing
Habitat isolé	Individual housing
Hippodrome	Unsuitable
hôtellerie	Unsuitable
Industrie	Unsuitable

KaruCover 2017 Use Class	New Use Class
Maraîchage et Autres cultures	Cropland
Maritime	Unsuitable
Parc tertiaire	Unsuitable
Parc urbain et espace vert	Unsuitable
Parking et Place	Unsuitable
Pêche et aquaculture	Unsuitable
Petit collectif mixé	Unsuitable
Petit collectif non mixé	Unsuitable
peuplement à potentiel sylvicole	Other
Production d'énergie éolienne	Other
Production d'énergie fossile	Unsuitable
Production d'énergie géothermique	Unsuitable
Production d'énergie hydraulique	Unsuitable
Production d'énergie photovoltaïque	Unsuitable
Résidentiel individuel faiblement compact (< 30 %)	Individual housing
Résidentiel individuel fortement compact (+ 80%)	Individual housing
Résidentiel individuel moyennement compact (30 et 80%)	Individual housing
Routier	Other
Sans usage	Other
Santé	Unsuitable
Serre et pépinière	Cropland
Service culturel et de divertissement	Unsuitable
Service religieux	Unsuitable
Transport et distribution d'électricité, de gaz, de pétrole	Other
Verger	Cropland
Zone de transition (chantier)	Unsuitable

S5 Table. Conversion of combined symplified cover & use classes to final landcover classes used for modeling

**Combined symplified cover & use classes**	**Final landcover class**
No vegetation ; Breeding	Unvegetated area
No vegetation ; Cropland	Unvegetated area
No vegetation ; Agricultural	Unvegetated area
No vegetation ; Other	Unvegetated area
No vegetation ; Unsuitable	Unvegetated area
No vegetation ; Dense housing	Unvegetated area
No vegetation ; Individual housing	Unvegetated area
Wetland ; Other	Wet area
Wetland ; Individual housing	Wet area
Wetland ; Agricultural	Wet area
Wetland ; Unsuitable	Wet area
Forest ; Unsuitable	Innaccessible area
Forest; Individual housing	Individual garden
Forest; Other	Forest
Forest; Agricultural	Agricultural area
Forest; Cropland	Cultivated area
Forest; Dense housing	Innaccessible area
Arable land ; Cropland	Cultivated area
Arable land ; Agricultural	Agricultural area
Arable land ; Other	Cultivated area
Thicket ; Cropland	Cultivated area
Thicket; Agricultural	Agricultural area
Thicket; Unsuitable	Innaccessible area
Thicket; Other	Thicket
Thicket; Individual housing	Individual garden
Thicket; Dense housing	Innaccessible area
Grass ; Breeding	Breeding area
Grass ; Unsuitable	Innaccessible area
Grass ; Agricultural	Agricultural area
Grass ; Other	Grass
Grass ; Cropland	Cultivated area
Grass ; Dense housing	Innaccessible area
Grass ; Individual housing	Individual garden

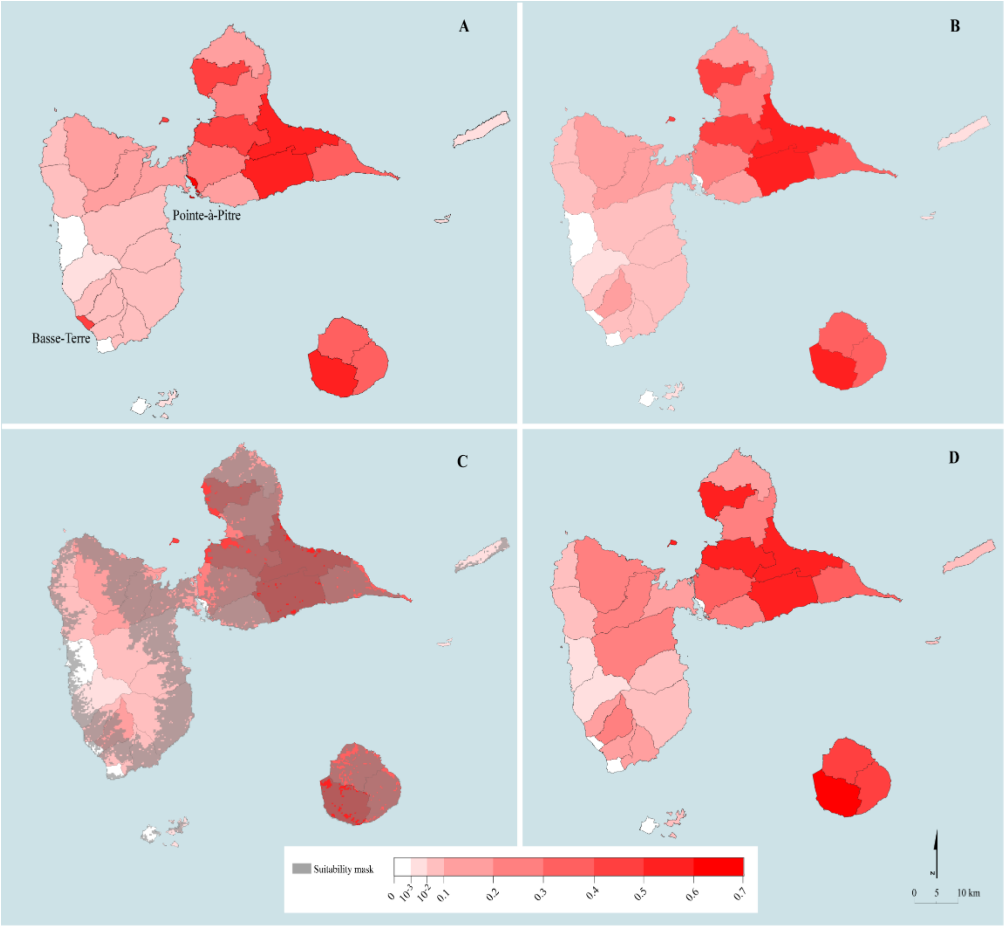

S6. Process of correcting the cattle density map: A) original map; B) redistribution of cattle densities from urban municipalities to neighbouring rural municipalities; C) map with superposition of the suitable areas mask; D) cattle density map with density corrected according to the surface of suitability. Created by Victor Dufleit using BD_TOPO® shapefile data. BD_TOPO® are open access data published by French National Geographic Service under "Etalab 2.0" licence.

The results of the redistribution of animals in urban area are shown in S6. This figure also illustrated the municipal cattle density recalculated based on the area of the suitable pixels. Pointe-à-Pitre city was originaly reported to have 171 animals. Those animals were redistributed to Les Abymes (original cattle count = 1754; after redistribution = 1901) and Gosier (original cattle count = 841; after redistribution = 865). 222 cattle were declared being in Basse-Terre city, they have been redistributed to Baillif (original cattle count = 179; after redistribution = 239), Saint-Claude (original cattle count = 315; after redistribution = 409) and Gourbeyre (original cattle count = 155; after redistribution = 223). The resulting density aftersuitability masking differ from original ones, particularly on Basse Terre Island (west), where natural areas constitute a large part of the landscape.

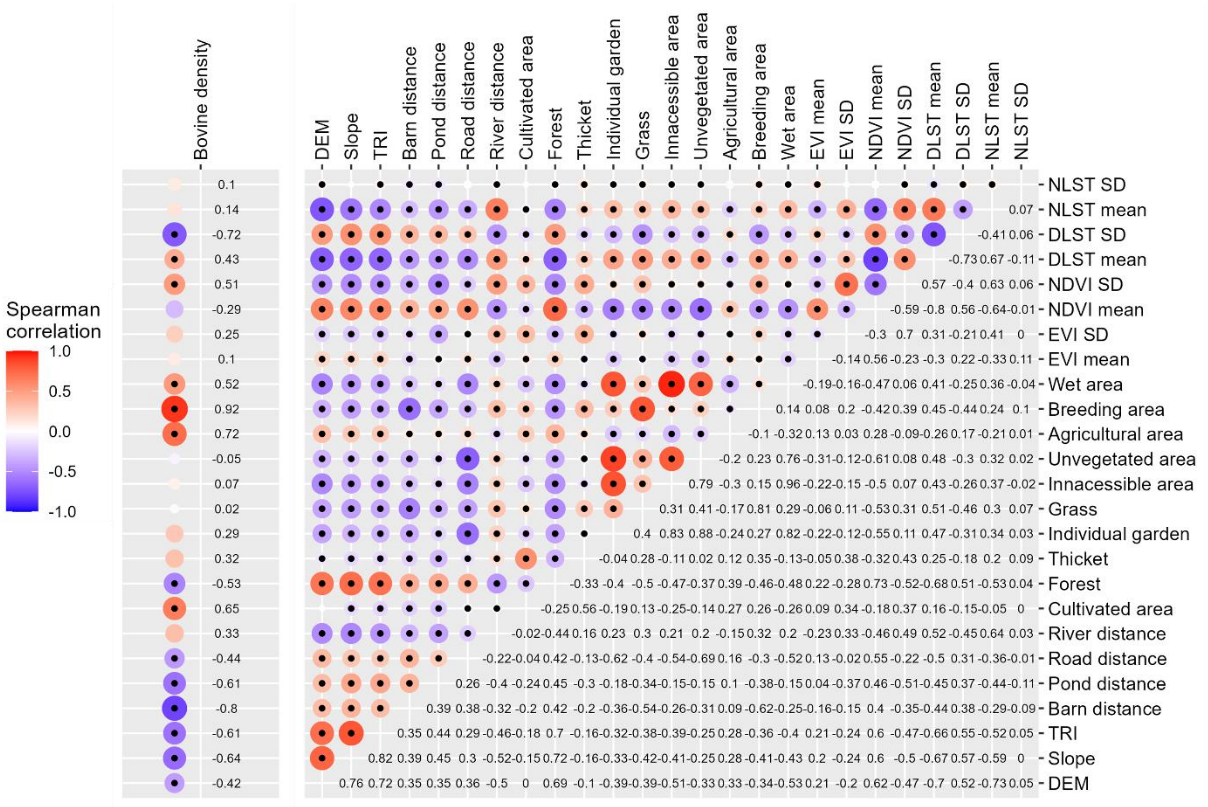

S7. Left: Spearman correlations between cattle density and predictors; right: correlations between predictors. The black dot indicates a significant correlation ($P < 0.05$); for clarity, predictors derived from the Fourier analysis of MODIS data are not presented, as the correlation between these variables and cattle density was difficult to interpret.
